# Rates, Risk Factors, and Progression of Diabetic Retinopathy in Children with Type 1 Diabetes: A 15-Year Retrospective Study from a Regional Center in New Zealand

**DOI:** 10.1155/2024/5893771

**Published:** 2024-06-28

**Authors:** Thomas Winter, José G. B. Derraik, Craig A. Jefferies, Paul L. Hofman, James A. D. Shand, Geoffrey D. Braatvedt, Stuti L. Misra

**Affiliations:** ^1^ Department of Ophthalmology New Zealand National Eye Centre Faculty of Medical and Health Sciences University of Auckland, Auckland, New Zealand; ^2^ Department of Paediatrics Child and Youth Health Faculty of Medical and Health Sciences University of Auckland, Auckland, New Zealand; ^3^ Environmental-Occupational Health Sciences and Non-Communicable Diseases Research Group Research Institute for Health Sciences Chiang Mai University, Chiang Mai, Thailand; ^4^ Department of Women's and Children's Health Uppsala University, Uppsala, Sweden; ^5^ Starship Child Health Health New Zealand, Te Whatu Ora Auckland, Auckland, New Zealand; ^6^ Liggins Institute University of Auckland, Auckland, New Zealand; ^7^ Department of Endocrinology Greenlane Clinical Centre Health New Zealand, Te Whatu Ora, Auckland, New Zealand

## Abstract

**Aims:**

Diabetic retinopathy (DR) is the primary microvascular complication associated with diabetes. Evidence on DR prevalence among children in New Zealand is scarce. We examined DR rates and associated risk factors in youth with type 1 diabetes (T1D) aged <16 years receiving care from a regional diabetes service in January 2006–December 2020.

**Materials and Methods:**

DR diagnosis followed the International Society for Pediatric and Adolescent Diabetes guidelines. The study included 646 participants; mean age (±SD) at T1D diagnosis was 7.4 ± 3.6 years, 47% were female, and 69% identified as NZ Europeans.

**Results:**

The initial DR screening occurred at a mean age of 12.6 ± 2.4 years and 5.2 ± 2.2 years after T1D diagnosis. At the first DR screen, 23.5% of participants (152/646) were diagnosed with DR: 69.1% (105/152) with minimal, 30.3% (46/152) with mild, and one moderate case (0.7%). Older age at diagnosis (*p*=0.029) and longer diabetes duration (*p*=0.015) were predictors of DR at first screen. Patients with at least one positive DR screen had a higher average HbA1c at their first screen (+2.6 mmol/mol; *p*=0.042). Overall, 55.6% (359/646) of patients had a positive DR screen, whose worst grade was mostly either minimal (58.2%) or mild (40.7%) DR, with only three moderate cases (0.8%) and one severe (0.3%). Children diagnosed with T1D before age 10 were 72% more likely to have DR than older children (*p* < 0.0001), and DR risk was 32% and 41% higher among Pacific children than NZ European (*p*=0.008) and Māori (*p*=0.014) children. Lastly, the only predictor of DR at discharge from paediatric services was HbA1c at the first screen (*p* < 0.0001).

**Conclusions:**

In this regional cohort of children with T1D, there was a high rate of low-grade DR overall and at first retinal screen, with an increasing rate until transfer to adult services. Our findings underscore the importance of ongoing DR screening, reducing glycaemic levels, and supporting vulnerable high-risk groups.

## 1. Introduction

Diabetes mellitus (DM) is associated with a range of debilitating complications. Diabetic retinopathy (DR) is the most common microvascular complication of DM globally [1]. In the developed world, DR is the leading cause of new-onset blindness among the working-age population [2].

Sight-threatening DR typically develops many years after the initial diagnosis of diabetes [3]. A robust body of evidence indicates that poor glycaemic control is a modifiable risk factor for the incidence and progression of DR [4, 5]. Landmark studies, including the Diabetes Control and Complications Trial (DCCT) and its follow-up (the Epidemiology of Diabetes Interventions and Complications—EDIC), have shown that tight glycaemic control in both youth and adulthood limits the development of sight-threatening DR [6, 7, 8]. Unfortunately, achieving good glycaemic control remains challenging for clinicians and young patients [9].

Although the evidence supporting tight glycaemic control in reducing diabetes complications is well-established, other important research areas remain underexplored. For example, evidence is less convincing regarding the right approach to DR screening protocols, and estimates of actual DR prevalence in children and young people vary markedly across studies [3, 4, 5, 10, 11, 12]. Changes in clinical management of diabetes can explain some of the variation in prevalence estimates. DR prevalence estimates from the 1980s (often over 40%, with mean patient ages under 18 years) likely reflect poorer glycaemic control in those study populations [10]. However, a review of the literature after 2010 revealed prevalence estimates ranging from 2.3% (mean age of 14.5 years) to 11.8% (mean age of 11.9) and up to 19.5% (median age of 14 years), with average HbA1c at screening in these populations of 67, 74, and 73 mmol/mol, respectively [4, 11].

Evidence on DR prevalence in children in New Zealand is limited. Previous studies have highlighted poor glycaemic control and low attendance at diabetes clinics and retinal screening appointments among children [12, 13]. Nonetheless, a recent study suggested rates of DR are decreasing, surprisingly, without marked concomitant improvements in glycaemic control [14].

This study aimed to examine the epidemiology of DR, including rates, risk factors, and progression among youth with type 1 diabetes (T1D) attending clinics at a regional diabetes center in Auckland, New Zealand.

## 2. Materials and Methods

### 2.1. Ethics

Ethics approval was provided by the Auckland District Health Board Research Review Committee (A + 6898). Written or verbal informed consent from patients was not required, as this study constituted an audit of routine clinical practice involving deidentified data.

### 2.2. Study Population

Clinical data were drawn retrospectively from the local diabetes database, which documents all children aged <15 years with T1D in the Auckland region, our single regional tertiary diabetes center. This paediatric service provides specialist care for our regional population of approximately 1.7 million as part of free universal healthcare. The diagnosis of T1D was made on clinical and biochemical features:

(1) elevated blood glucose at presentation (random measurement >11.1 mmol/L and/or fasting sample >7.1 mmol/L),
(2) classic symptoms of diabetes, and
(3) meeting at least one of the following criteria:
  - Diabetic ketoacidosis.
  - Presence of T1D-associated antibodies.
  - Ongoing requirements for insulin therapy.

Additional inclusion criteria for this study are as follows:

- T1D diagnosis before 1 January 2015.
- Having attended at least one diabetes clinic at Starship Children's hospital between 1 January 2006 and 31 December 2020.
- Aged <16 years at the diabetes clinic.
- At least one DR screen between 1 January 2006 and 31 December 2020.
- Aged < 17.0 years at DR screen.

### 2.3. Retinopathy Screening and Grading

Retinopathy screening data for the included children were extracted from a review of patient notes and integrated with the most recent preceding diabetes clinic visit. DR screening was performed using fundus photography according to the New Zealand Ministry of Health guidelines [15]. Retinopathy was examined at age 10, or from 5 years after the diabetes diagnosis, whichever occurred first, based on International Society for Pediatric and Adolescent Diabetes (ISPAD) Clinical Practice Consensus Guidelines [16]. Overall grades (none, minimal, mild, moderate, severe, or proliferative) were assigned by trained personnel, including experienced optometrists and specialist diabetes nurses with specific training on DR screening, at either Starship Children's Hospital or the Greenlane Clinical Centre. Any children with moderate or severe retinopathy grades had their images additionally rechecked by an ophthalmologist, with any amendment designated as the most appropriate grade and used in the database.

### 2.4. Other Demographic and Clinical Data

Information extracted from the database included the participant's age at T1D diagnosis, biological sex, and caregiver-reported ethnicity. The latter was stratified using a hierarchical classification system, with patients assigned to one of four categories in the following prioritized order: Māori, Pacific, Other, or NZ European [17]. Socioeconomic deprivation was assessed with the New Zealand Index of Multiple Deprivation 2018 (IMD18), a comprehensive area-based measure derived from 6,181 nationally ranked data zones, each with an average population of 761 [18, 19]. The IMD18 is derived from scores across seven different domains of deprivation: income, employment, crime, housing, health, education, and geographical access [18, 19].

Clinical data included glycated hemoglobin (HbA1c) and anthropometry at the immediate clinic visit preceding a given retinopathy screening. Weight, height, and body mass index (BMI) were transformed into standard deviation scores (SDS) adjusted for age and sex according to WHO standards [20].

The time-weighted (TW) HbA1c over 1 year was calculated for each participant with two or more HbA1c measurements within 365 days prior to a target screening visit:

(1) $$\begin{array}{c} \text{TW HbA1c}=\frac{\text{AUC}}{{\text{Date}}_{\text{t}}-{\text{date}}_{0}}, \end{array}$$

where AUC is the area under the curve, and date_0_ and date_*t*_ are the dates when the first HbA1c and last HbA1c measurements were made, respectively, with the difference expressed in days [21].

### 2.5. Statistical Analyses

The demographic and clinical characteristics of included and excluded patients were compared with one-wayANOVA for continuous variables and Fisher's exact tests for categorical variables. Continuous data are reported as mean ± standard deviation (SD), median (quartile 1, quartile 3), or the range, and categorical variables as frequency (*n*) and percentage (%). Appropriate pairwise comparisons between two rates were carried out using two-sample Poisson rate tests.

The associations between clinical and demographic parameters with the likelihood of retinopathy at the participant's first screen were examined using generalized linear regression models using a modified Poisson procedure, and expressed as the adjusted relative risk (aRR) and the respective 95% confidence interval (CI) [22]. Models adjusted for ethnicity (NZ European/Māori/Pacific/Other), sex (male/female), age group at T1D diagnosis (<10 years/≥10 years), socioeconomic deprivation (IMD18), HbA1c at the screen, and the duration between T1D diagnosis and the DR screen.

The same multivariable models were run to evaluate the risk of retinopathy at any point during the study period and specifically at discharge from the paediatric services. However, for the analysis at discharge, we only included data from participants who had undergone at least two screenings for retinopathy.

In these models, age at T1D diagnosis was stratified into a binary variable to minimize the multicollinearity issue due to its strong correlation with the duration from diagnosis to screen (*r* = −0.73; *p*  < 0.0001). Similarly, HbA1c and TW-HbA1c were not included in the same model (*r* = 0.88; *p*  < 0.0001), and each multivariable model was run separately with either variable, with HbA1c invariably selected for inclusion based on model fit, assessed using the generalized estimating equations (GEE) fit criteria, specifically the quasi-likelihood under the independence model criterion (QIC) [23].

The longitudinal progression of DR was visually represented, as the marked variability in the number of DR screens per patient limited the robustness of statistical analyses. Data from patients who underwent at least two DR screens during the audit period were categorized into 1-year brackets starting from the initial screening. For patients with multiple screens within a given 1-year period, only the last screen was considered.

Statistical analyses were conducted using SAS v9.4 (SAS Institute, Cary, NC, USA), including the calculations of AUC and the TW-HbA1c. All tests were two-sided with significance set at *p* < 0.05. The progression of DR rates and severity over time were derived using algorithms and illustrated in Microsoft Excel for Office 365 v2404 (Microsoft Corporation, Redmond, WA, USA). Other graphs were created in GraphPad Prism v8.4.3 (GraphPad Software Inc., San Diego, CA, USA).

## 3. Results

### 3.1. Study Population

There were 1,144 paediatric patients with diabetes in the Auckland City Hospital “Starbase” with recorded clinic visits during the 15-year study period (2006–2020). Our study included 646 youth with T1D who underwent at least one DR screen, after excluding 235 patients with other forms of diabetes, 29 without clinic visits at age <16 years, and 234 without any recorded DR screen (Figure S1).

The demographic characteristics of included and excluded patients are shown in Table S1. Included participants were diagnosed, on average, 2 years earlier and were 1.4 years younger at their first clinic visit recorded during the audit period (Table S1). Additionally, included participants had a lower representation of Māori and Pacific ethnicities, who were more likely to be excluded due to missing DR screening than patients from other reported ethnicities (66/176 (37%) vs. 197/730 (27%); *p*=0.038).

### 3.2. First Retinopathy Screening

At the first DR screen, the mean age was 12.6 years, with the youngest recorded screening at 5.9 years (Table S2). On average, the first DR screen occurred 5.2 years after T1D diagnosis; half of our patients were screened after 3.5–6.2 years, but the timing varied widely from 11 months to 13.7 years (Table S2). Nearly three of four patients (72%) underwent two or more DR screens, but screening frequency was markedly variable, with one patient screened 10 times during the audit period (Table S2).

### 3.3. Retinopathy Rates and Risk Factors

At their first recorded screen, 24% of patients (152/646) were diagnosed with DR (Table 1). Of these, 69% (105/152) had minimal and 30% (46/152) mild DR, with only one case (0.7%) of moderate severity (Table 1).

Patients who were diagnosed with DR at the first screen were, on average, 6 months older (*p*=0.025), experienced greater levels of socioeconomic deprivation (*p*=0.006), and had TW-HbA1c 3.5 mmol/mol higher than those without it (95% CI: 0.9, 6.1 mmol/mol; *p*=0.008) (Table 1). Multivariable analysis revealed that older age at T1D diagnosis (*p*=0.029) and a longer duration since T1D diagnosis (*p*=0.015) were predictors of DR at the first screen. Patients aged ≥10 years at T1D diagnosis had a DR risk 57% higher than younger counterparts (aRR = 1.57; 95% CI: 1.05, 2.37). After T1D diagnosis, every additional year of diabetes duration increased the DR risk at the first screen by approximately 10% (aRR = 1.10; 95% CI: 1.02, 1.19).

Over the 15-year study period, DR was diagnosed in 55.6% of patients (359/646), with the cumulative rates depicted in Figure S2. The median age for the initial DR diagnosis was 13.8 years (Table 1). While 75% of the children diagnosed with DR were 12 years or older, diagnoses were made in children as young as 5.9 years (Table 1). Nearly half (42%) of those with a positive DR screen received their diagnosis at their first DR screen (152/359) (Table 2).

The interval from T1D diagnosis to the first screening was only marginally shorter for those diagnosed with DR (≈5 months; *p*=0.022) (Table 3). DR rates were similar in males and females (Table 3). However, those with DR had been diagnosed with T1D 1.7 years earlier (*p* < 0.0001) and were 1.3 years younger (*p* < 0.0001) with slightly higher average HbA1c levels (+2.6 mmol/mol (95% CI: 0.1, 5.0 mmol/mol); *p*=0.042) at the time of their first screen (Table 3). Notably, Pacific participants were disproportionately represented among those with DR, with rates threefold higher than their counterparts (12% vs. 4%; Table 3), likely reflecting poorer glycaemic control compared to other ethnicities (mean TW-HbA1c at first screen 82.8 ± 17.8 vs 67.9 ± 13.1 mmol/mol; *p* < 0.0001).

In the multivariable analysis, age at T1D diagnosis and Pacific ethnicity were the only predictors of having DR at least once. Children diagnosed with T1D before age 10 were 72% more likely to develop DR than older children (aRR = 1.72 (95% CI: 1.37, 2.15); *p* < 0.0001). Pacific patients had a 32% higher DR risk compared to NZ Europeans (aRR = 1.32 (95% CI: 1.08, 1.63); *p*=0.008) and 41% higher than Māori (aRR = 1.41 (95% CI: 1.07, 1.85); *p*=0.014).

### 3.4. Retinopathy Severity

At the first diagnosis, most patients had DR graded as either minimal (70%; 252/359) or mild (29%; 105/359), with only two moderate cases (0.6%) and none severe (Table 2). When considering the participant's worst DR grade recorded, the classifications remained predominantly minimal (58%) or mild (41%), with only three moderate cases (0.8%) and one severe (0.3%) (Table 2). The demographic and clinical characteristics of the four patients with moderate or severe DR did not reveal any consistent markers (Table S3).

When comparing participants by their worst grades, those with minimal and mild DR had similar sex and ethnicity distributions, yet patients with mild DR experienced higher levels of socioeconomic deprivation (*p*=0.018; Table S4). Children with mild DR were diagnosed with T1D ≈ 10 months earlier (*p*=0.023) but underwent their first DR screening ≈ 14 months later (*p*=0.0001) (Table S4). Children with mild DR had an average HbA1c 5.2 mmol/mol higher at their screening visit (95% CI: 1.8, 8.6 mmol/mol; *p*=0.003) and a TW-HbA1c 4.9 mmol/mol higher over the previous year (95% CI: 1.8, 8.0 mmol/mol; *p*=0.002), indicating an association between glycaemic control and DR severity (Table S4).

### 3.5. Retinopathy Progression

**Figure 1:**
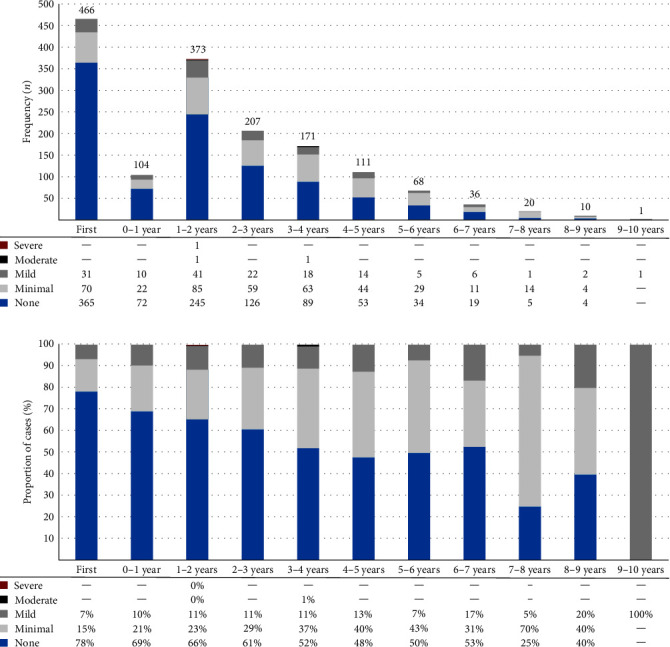
Diabetic retinopathy (DR) rates and severity among children and adolescents with type 1 diabetes mellitus (T1D) in the Auckland region, based on time elapsed since their initial DR screening during the audit period (2006–2020). Inclusion was limited to patients with ≥2 DR screenings (*n* = 466) to assess changes in DR severity over time. Each bar in (a, b) represents a 1-year bracket, with “0–1 year,” for example, indicating a follow-up screening within 1 year from the first. For patients with multiple screenings within a given 1-year period, only the last screening was considered: (a) shows the total screenings and distribution of DR severity at the last screening for each period; (b) illustrates the proportions of these screenings by DR severity. Severity categories were defined based on ISPAD Clinical Practice Consensus Guidelines.

Figure 1 illustrates DR rates among the 466 youths with two or more recorded screenings during the study, according to the time elapsed from their first screening. The proportion of patients without DR appeared to decrease over time from 78% at the first screening to approximately 50% three or more years later. Conversely, there appears to have been a corresponding increase in the proportion of patients with minimal DR, which rose from 15% at the first screening to 30%–40% 3 or more years later (Figure 1(b)). However, the number of patients screened beyond 5 years from the initial screening was relatively small for reliable inferences about long-term DR progression in this cohort (Figure 1(a)).

Among the same patients, 42% (196/466) were diagnosed with DR at their final paediatric service screening. HbA1c level at the first screening was the sole predictor of DR at transition, with the risk increasing by approximately 10% for every 5 mmol/mol rise in HbA1c (aRR = 1.10 (95% 1.06, 1.14); *p* < 0.0001). Among patients diagnosed with DR at any time who also underwent multiple screenings (*n* = 359; Table 2), 36.7% (*n* = 113) showed no DR signs at their final paediatric screening.

### 3.6. Retinopathy Screening Guidelines

**Figure 2:**
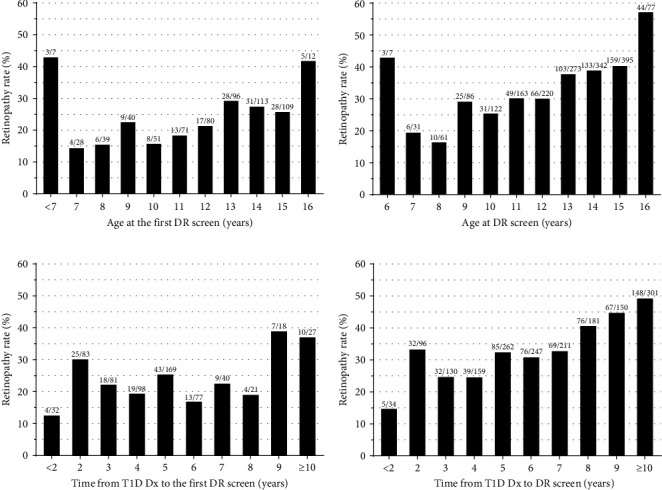
Diabetic retinopathy (DR) rates among 646 children and adolescents with type 1 diabetes (T1D) in the Auckland region over 15 years (2006–2020). Panels show DR rates according to (a) age at first DR screen (*n* = 646); (b) age at any recorded DR screen (*n* = 1,777); (c) time elapsed from T1D diagnosis (Dx) to first DR screen (*n* = 646); and (d) time elapsed from T1D Dx to all recorded DR screens (*n* = 1,777).

Our analysis of the current DR screening guidelines for paediatric patients with T1D, which recommend starting screenings at either age 10 or 5 years after T1D diagnosis (whichever occurs first) [15, 16] revealed nuanced findings. Excluding the small outlier group of very young children (<7 years) with a high diagnosis rate (3/7; 43%), we observed a relatively gradual increase in DR rates from age 10 without a clear threshold for escalation (Figure 2(a)). For example, the rate was 23% at age 9 years (9/40), which was comparable to 22% at age 12 (17/80) and 26% at age 15 (28/109) (Figure 2(a)). When considering all screenings (*n* = 1,777), we observed a gradual increase in DR rates starting from ages 7 to 8 (Figure 2(b)), although repeated screenings of individuals previously diagnosed could have skewed the data.

The assessment of diabetes duration showed no marked increase in DR rates at the 5-year mark (Figure 2(c)). Instead, a 2-year postdiagnosis period emerged as a potential risk threshold, with a 30% positive screening rate (25/83) compared to 25% (43/169) for those first screened at 5 years (Figure 2(c)). In addition, there was a consistent increase in detection rates over time across all screenings, yet this trend did not substantiate the 5-year mark as a pivotal threshold for increased DR risk (Figure 2(d)).

**Figure 3:**
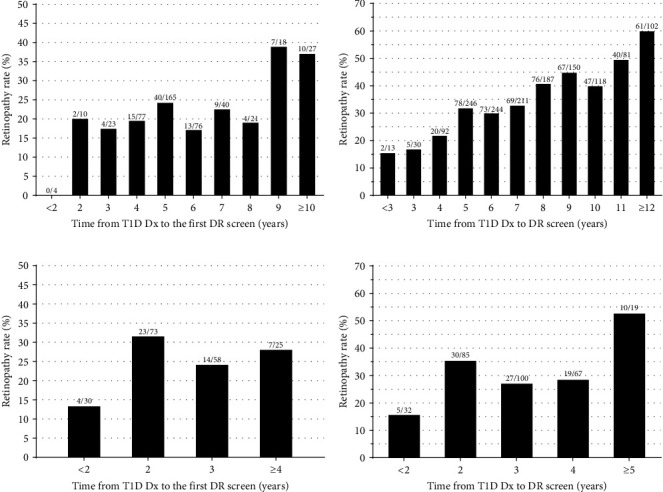
Diabetic retinopathy (DR) rates among 646 children and adolescents with type 1 diabetes (T1D) in the Auckland region over 15 years (2006–2020), according to age at diagnosis (Dx) and the time elapsed from T1D Dx to DR screen. Panels show DR rates according to age at (a) first DR screen in children <10 years at T1D Dx (*n* = 460); (b) any recorded DR screen in children aged <10 years at T1D Dx (*n* = 1,474); (c) first DR screen in children ≥10 years at T1D Dx (*n* = 186); and (d) any recorded DR screen in children ≥10 years at T1D Dx (*n* = 303).

The evaluation of DR rates against the guidelines' key criteria indicated no distinct thresholds for children diagnosed with T1D before age 10 (Figures 3(a) and 3(b)). While there were increased rates at 2–3 years and at ≥9 years after T1D diagnosis, these were periods with fewer screenings (Figure 3(a)). Conversely, for children diagnosed at ≥10 years, there was a marked rise in DR rates at the 2-year mark (32%; 23/73), a stark contrast to the 13% rate (4/30) before 2 years (Figure 3(c)). This pattern persisted across all screenings for this age group, with a noted spike in DR incidence after 5 years, albeit based on fewer screenings (53%; 10/19) (Figure 3(d)).

### 3.7. Summary of Key Metrics

Overall, the data recorded in this study paints a complex picture among our paediatric patients with T1D in the Auckland region. Key DR metrics (including risk factors) are therefore summarized in Table 4.

## 4. Discussion

The current study, conducted in New Zealand's largest regional center, showed a DR rate (of any severity) of 55.6% among all patients over 15 years. This is a substantial figure that reflects the genuine risk of onset and progression of microvascular complications of T1D, even in children and adolescents. This rate also suggests that timely and appropriate screening of DR is important in preventing one of the most debilitating long-term sequelae of T1D. In contrast, the rate at the first retinopathy assessment was 23.5%, comparatively higher than recent data and estimates from other countries and New Zealand centers [11, 12, 24, 25]. The reasons behind this discrepancy are currently unclear. Further, given the potential for background DR to regress, the DR rate across the 15 years may be deceptively high. Nonetheless, only two patients (0.6% of those with DR at the first screen) exhibited DR greater in severity than mild at the first screen (both moderate). Indeed, across all screening data, only four patients were diagnosed with DR of moderate (*n =* 3) or severe (*n =* 1) grades.

This study revealed generally poor glycaemic control within our study population. The mean values for HbA1c were 71.8 ± 15.0 and 69 ± 16.1 mmol/mol in those with and without retinopathy at the first screen, respectively. These figures are markedly higher than clinically recommended levels, such as the target HbA1c of <53 mmol/mol recommended for youths by the American Diabetes Association [26]. Similar findings from a New Zealand study noted patients aged <15 years had a median HbA1c of 70 mmol/mol in 2019, and in 2017, those under 16 had a mean HbA1c of 71 ± 3 mmol/mol [14, 27]. In a multicenter Australasian study, the mean HbA1c in those aged 10–15 was 66 mmol/mol in 2019 [28].

The link between poor glycaemic control and worse microvascular outcomes has been well-established since the 1990s and is reaffirmed here. In patients with DR, HbA1c at the preceding clinic visit was higher in those with worse retinopathy. In those patients with more than one retinopathy screening, DR rates increased by approximately 9% for each 5 mmol/mol increase in HbA1c at the first screen. Given our observed poor glycaemic control and its association with DR risk, improving glycaemic control in children and adolescents in New Zealand is important. Technologies such as continuous glucose monitoring (CGM) and continuous subcutaneous insulin infusion (CSII) markedly improve glycaemic control and reduce microvascular complications in youth [29, 30].

Studies have shown that CGM results in better glycaemic control than standard blood glucose (finger-prick) monitoring [31, 32]. However, others noted that the benefits of CGM were less pronounced in children [33, 34], with several challenges in integrating CGM data into daily management. Despite these challenges, there is increasing evidence that CGM improves glycaemic control in children and adolescents with T1D, reducing the risk of hypoglycaemia and diabetic ketoacidosis [35, 36, 37, 38, 39]. There is also consistent evidence that insulin pumps improve glycaemic control and reduce total daily insulin dose in children and adolescents with T1D [29, 40]. Thus, insulin pumps are associated with lower risks of severe hypoglycaemia and diabetic ketoacidosis [40, 41, 42]. However, to our knowledge, no studies have yet assessed the impact of insulin pump therapy on the DR risk in paediatric patients. Therefore, increasing access to these technologies will likely lead to marked patient benefits. Such technologies also provide more equitable health outcomes for young people [14, 27, 28, 37].

New Zealand's social security system offers free medical care [43]. The public healthcare system aims to provide equitable access to all necessary medical services for children and adolescents [43], irrespective of private insurance coverage. Essential diabetes management services, including monitoring and treatment, are freely available to this demographic, with minimal direct costs of T1D for patients' families [44]. Of note, no private health insurance in the country covers any aspect of T1D care, including CGM, pump therapy, or analog insulins. Therefore, income and insurance coverage in New Zealand is unlikely to directly affect access to and quality of T1D care for children and adolescents [44], and consequently, glycaemic control and the associated DR risk in this age group. Nonetheless, we observed disparities in health outcomes, suggesting that factors beyond simple access to care contribute to these inequities. In particular, Pacific ethnicity and higher levels of socioeconomic deprivation were identified as factors associated with increased DR risk. Since Pacific people are more likely to experience material hardship, low incomes, and housing deprivation [45, 46], their higher DR rate could be a consequence of socioeconomic disparities and their adverse impact on glycaemic control. However, the multivariable model, including a measure of socioeconomic deprivation (IMD18), showed that Pacific ethnicity was the stronger predictor. Moreover, while Pacific patients experienced higher deprivation levels, these were no different from Māori patients (Figure S3), whose DR rates were lower. Therefore, besides greater socioeconomic deprivation, other associated factors may include perceptions of institutional racism, a reluctance to engage with mainstream medical services, and language barriers. As a result, the observed disparities require a comprehensive investigation into the underlying factors to improve service delivery and achieve equitable outcomes, including reducing DR rates.

The relatively high DR rates at first screening (23.5%) and overall (55.6%) suggest that the screening and clinical management of paediatric patients with T1D must be improved. Current screening guidelines in New Zealand specify that children newly diagnosed with T1D should be screened for DR within 5 years or at age 10, whichever comes first [15]. This guideline is based on ISPAD Clinical Practice Consensus Guidelines from 2014, which recommend commencement of screening “at age 10 or at the onset of puberty if this is earlier, after 2 to 5 years' diabetes duration” [16]. The analysis performed here provides some evidence supporting these thresholds. However, our study did not identify a substantial increase in DR rates at the 5-year mark following diagnosis. Conversely, there was weak evidence of an earlier threshold 2 years after diagnosis. While it is likely that clinical factors might have triggered early screening, our study provides some support for an earlier time point for DR screening after T1D diagnosis.

Additionally, more than one-quarter of patients (234/880; 27%) were excluded from the analyses because they had no recorded retinopathy screening. While some of these patients may have undergone screening photographs in private clinics, many were likely never screened. These patients were more likely to be Māori or Pacific, complementing existing evidence that inequitable barriers (such as access to transport, inadequate booking systems, and lack of engagement with patients and their families) limit access to retinopathy screening for Māori and Pacific [13, 47]. In addition, our paediatric diabetes and retinal screening services in Auckland are not restricted to a single central clinic. Rather, we have clinics at four different sites in the region, so diabetes care is accessible within a 30-min drive of any patient with T1D living in Auckland. Nevertheless, in light of the observed ethnic and socioeconomic disparities in DR rates and other diabetes-related comorbidities, some researchers have argued for a shared-care model involving community-based optometrist screening to improve access [13]. Further research is essential to more accurately characterize screening barriers and mitigate inequities in access to DR screening.

Other study limitations include its retrospective and audit nature, hence the wide variation in timing and frequency of the retinal screens. Additionally, some patients' actual first retinopathy screens could have been excluded if they had taken place before 2006 or if they had attended a private screening clinic. Precise evaluation of DR progression in the long-term (beyond 5 years since the first DR screening) was hindered by the decreasing number of patients captured undergoing screening at longer intervals, though this would be expected since paediatric patients transition to adult diabetes services at age 15. We also could not account for the use of CGM or insulin pumps. In New Zealand, nationwide data from 2021 reported marked inequities in CGM access by ethnicity and socioeconomic deprivation [37]. However, our audit period ended in December 2020, and CGM only became available for paediatric patients with T1D in this country in March 2018 [48]. Thus, CGM access would likely have had a minimal impact (if any) on our findings, as initial CGM uptake in New Zealand was very limited. Regarding insulin pumps, these have been publicly funded in New Zealand since 2012, and their uptake progressively increased over the years covered by this study [49, 50]. Still, by 2016, only 25% of paediatric patients nationwide were reportedly using them, with both ethnic and socioeconomic disparities in uptake [49, 50]. Nonetheless, pump therapy might have been a factor underlying lower DR rates in some patients, which could explain, at least in part, some of our observed ethnic differences. Notably, our findings corroborate previous New Zealand data showing that ethnicity and socioeconomic status were independently associated with poorer glycaemic control and increased risk of diabetes-related comorbidities, highlighting the complexity of health inequities consistently reported in Pacific and Māori patients [51]. Furthermore, as mentioned earlier, Pacific and Māori patients were more likely to have been excluded due to a lack of retinopathy screening, a possible source of selection bias. Differences in glycaemic control were not statistically significant between included and excluded patients. However, given the evidence of poorer outcomes for Pacific and Māori patients in other studies on DR in New Zealand, the rate of DR may have been underestimated [13, 47]. Lastly, with the increasing use of insulin pumps and particularly CGM, greater access to such technologies would likely impact DR rates and severity. Future studies should examine the complex interactions between these factors to ascertain their impacts on DR rates and identify mitigating measures to address existing ethnic and socioeconomic disparities in diabetes management and outcomes.

## 5. Conclusions

The DR rate at first screening was notably high in the Auckland region, with over half of the patients experiencing DR at least once, underscoring the need for improved long-term glycaemic control to prevent microvascular complications. Some evidence supported the current duration- and age-based screening thresholds. While it was difficult to draw best-practice conclusions about the precise timing of DR screening, conducting DR screenings for children yielded clinically valuable information. Poor glycaemic control and the duration of T1D were reconfirmed as key DR predictors. Additionally, inequitable DR patterns were noted to adversely affect Pacific patients; therefore, barriers to improve glycaemic control and screening access must be addressed. Further research is essential to clarify the underlying factors leading to inequities in diabetes care and health outcomes and to understand why many children miss DR screenings.

## Figures and Tables

**Table 1:** Demographic and clinical characteristics of children and adolescents with type 1 diabetes mellitus (T1D) in the Auckland region at their first recorded diabetic retinopathy (DR) screen in the study period.

Characteristic	Parameter	Levels	No retinopathy	Retinopathy	*p*-Value
*n*	—	—	494 (76.5%)	152 (23.5%)	—

Demography					

	Sex	Female	228 (46%)	74 (49%)	0.64
		Male	266 (54%)	78 (51%)	

	Ethnicity	NZ European	350 (71%)	96 (63%)	0.15
		Māori	44 (9%)	15 (10%)	—
		Pacific	35 (7%)	19 (13%)	—
		Other	65 (13%)	22 (14%)	—

	IMD18^*∗*^	—	4 (2, 7)	5 (3, 8)	**0.006**

Clinical					

	Height SDS	—	0.51 ± 0.99	0.44 ± 1.04	0.46
	Weight SDS	—	0.74 ± 0.89	0.78 ± 0.87	0.15
	BMI SDS (kg/m^2^)	—	0.64 ± 0.81	0.73 ± 0.80	0.26

	Age at T1D Dx (years)	—	7.4 ± 3.5	7.6 ± 3.6	0.44
		—	7.1 (4.6, 10.4)	7.6 (4.9, 10.4)	—
		—	0.8–14.5	0.7–14.2	—

	Age at DR screen (years)	—	12.5 ± 2.5	13.0 ± 2.4	**0.025**
		—	12.9 (10.8, 14.5)	13.5 (11.5, 14.9)	—
		—	6.5–16.4	5.9–16.2	—

	Time from T1D Dx (years)	—	5.1 ± 2.1	5.4 ± 2.5	0.22
		—	5.1 (3.5, 6.1)	5.2 (3.4, 6.3)	—
		—	0.9–12.3	1.5–13.7	—

	DR screens (*n*)	1	129 (26%)	51 (34%)	0.059
		2	134 (27%)	41 (27%)	—
		3	75 (15%)	30 (20%)	—
		4	70 (14%)	15 (10%)	—
		5	43 (9%)	5 (3%)	—
		≥6	43 (9%)	10 (7%)	—

	HbA1c at screen (mmol/mol)	—	69.0 ± 16.1	71.8 ± 15.0	0.053

	Time-weighted HbA1c (mmol/mol)	—	68.4 ± 14.2	71.9 ± 13.9	**0.008**

Continuous data are the mean ± standard deviation, the median (quartile 1, quartile 3), or the range; categorical data are reported as *n* (%). BMI, body mass index; Dx, diagnosis; HbA1c, glycated hemoglobin; IMD18, 2018 New Zealand Index of Multiple Deprivation; NZ, New Zealand; SDS, standard deviation score; and T1D, type 1 diabetes mellitus.  ^*∗*^The IMD18 is an area-based measure of socioeconomic deprivation where higher scores represent higher deprivation levels; data were available for all but two and one patients with and without retinopathy at their first screen, respectively. *p*-Values were derived from one-way ANOVA, nonparametric Kruskal–Wallis test, or Fisher's exact tests; statistically significant *p*-values (at *p* < 0.05) are highlighted in bold.

**Table 2:** Timing and grading of diabetic retinopathy (DR) among children and adolescents with type 1 diabetes in Auckland.

Parameter	Levels	*n* (%)
Diagnosed with DR	—	359 (55.6%)

Age at first DR diagnosis (years)	—	13.3 ± 2.2
	—	13.8 (12.0, 15.0)
	—	5.9–16.7

DR diagnosed at first screen	Yes	152 (42.3%)
	No	207 (57.8%)

DR grade at first screen^†^	Minimal	105 (69.0%)
	Mild	46 (30.3%)
	Moderate	1 (0.7%)

First DR grade overall	Minimal	252 (70.2%)
	Mild	105 (29.2%)
	Moderate	2 (0.6%)

Worst DR grade overall	Minimal	209 (58.2%)
	Mild	146 (40.7%)
	Moderate	3 (0.8%)
	Severe	1 (0.3%)

DR screen result at the last clinic visit	No retinopathy	113 (36.7%)
	Minimal	170 (47.4%)
	Mild	72 (20.1%)
	Moderate	3 (0.8%)
	Severe	1 (0.3%)

Age data are the mean ± standard deviation, the median (quartile 1, quartile 3), and the range; categorical data are reported as *n* (%). ^†^The denominator is the number of patients diagnosed with DR at their first screen (*n* = 152).

**Table 3:** Demographic and clinical characteristics of children and adolescents with type 1 diabetes in Auckland who were screened for diabetic retinopathy, comparing those who were and were not diagnosed with retinopathy at least once during the study period (2006–2020).

Characteristic	Parameter	Levels	No retinopathy	Retinopathy	*p*-Value
*n*	—	—	287 (44%)	359 (56%)	—

Demography	Sex	Female	134 (47%)	168 (47%)	>0.99
		Male	153 (53%)	191 (53%)	—
	Ethnicity	NZ European	208 (73%)	238 (66%)	**0.005**
		Māori	29 (10%)	29 (8%)	—
		Pacific	12 (4%)	43 (12%)	—
		Other	38 (13%)	49 (14%)	—
	IMD18^*∗*^	—	4 (2, 7)	4 (2, 7)	0.12
	Height SDS^†^	—	0.51 ± 0.98	0.48 ± 1.02	0.66
	Weight SDS^†^	—	0.81 ± 0.84	0.70 ± 0.92	0.15
	BMI SDS (kg/m^2^)^†^	—	0.69 ± 0.78	0.64 ± 0.72	0.37

Clinical	Age at T1D Dx (years)	—	8.4 ± 3.5	6.7 ± 3.4	**<0.0001**
		—	9.0 (5.6, 11.4)	6.4 (3.8, 9.4)	—
		—	1.1–14.7	0.7–14.2	—
	Age at screen (years)^†^	—	13.3 ± 2.2	12.0 ± 2.5	**<0.0001**
		—	14.1 (11.9, 15.2)	12.3 (10.2, 14.0)	—
		—	7.2–16.4	5.9–16.2	—
	Time from T1D Dx (years)	—	5.0 ± 2.2	5.4 ± 2.2	**0.022**
		—	4.9 (3.2, 6.1)	5.2 (4.0, 6.3)	—
		—	1.0–12.3	0.9–13.7	—
	Retinopathy screens (*n*)	1	129 (45%)	51 (14%)	**<0.0001**
		2	97 (34%)	78 (22%)	—
		3	28 (10%)	77 (21%)	—
		4	20 (7%)	65 (18%)	—
		5	9 (3%)	39 (11%)	—
		≥6	4 (1%)	49 (14%)	—
	HbA1c (mmol/mol)^†^	—	68.2 ± 16.4	70.8 ± 15.4	**0.042**
	Time-weighted HbA1c (mmol/mol)^†^	—	68.3 ± 15.0	69.9 ± 13.4	0.13

Continuous data are the mean ± standard deviation, the median (quartile 1, quartile 3), or the range; categorical data are reported as *n* (%). BMI, body mass index; Dx, diagnosis; HbA1c, glycated hemoglobin; IMD18, 2018 New Zealand Index of Multiple Deprivation; NZ, New Zealand; SDS, standard deviation score; and T1D, type 1 diabetes mellitus.  ^*∗*^The NZ IMD is a measure of socioeconomic deprivation where higher scores represent higher deprivation levels; data were available for all but two and one of patients who were and were not diagnosed with retinopathy at any time during the audit period, respectively. ^†^Parameters recorded at their first screen in the database that met the inclusion criteria for this study. *p*-Values were derived from one-way ANOVA, nonparametric Kruskal–Wallis tests, or Fisher's exact tests; statistically significant *p*-values (at *p* < 0.05) are highlighted in bold.

**Table 4:** Summary of key diabetic retinopathy (DR) metrics and associated risk factors among children and adolescents with type 1 diabetes (T1D) in the Auckland region.

Metric	Levels	Value/parameter
Overall DR rate during the 15-year study period	—	55.6% (359/646)

DR rate at first screening	—	23.5% (152/646)

Median age at initial DR diagnosis (years)	—	13.8 (5.9–16.7)

Proportion of DR diagnosed at first screening	—	42.3% (152/359)

Worst DR grade recorded	Minimal	58.2% (209/359)
	Mild	40.7% (146/359)
	Moderate	0.8% (3/359)
	Severe	0.3% (1/359)

Key risk factors for DR^*∗*^	—	Age at T1D diagnosis
	—	Duration of diabetes
	—	HbA1c levels
	—	Pacific ethnicity

DR rate at final paediatric screening^*∗*^	—	42.1% (196/466)

Sole predictor of DR at transition to adult care	—	HbA1c at first screening

Appropriate data are presented as percentages (*n*/*N*) or median (range). DR, diabetic retinopathy; HbA1c, glycated hemoglobin; T1D, type 1 diabetes mellitus.  ^*∗*^Based on multivariable generalized linear regression models.

## Data Availability

The dataset for this study, consisting of confidential and sensitive clinical audit information, cannot be made publicly available in compliance with New Zealand's Health and Disability Ethics Committees' Standard Operating Procedures (version 3.0).

## Abbreviations

aRR: adjusted relative risk
AUC: Area under the curve
BMI: Body mass index
CGM: Continuous glucose monitoring
CI: Confidence interval
DR: Diabetic retinopathy
HbA1c: Glycated haemoglobin
IMD18: 2018 New Zealand Index of Multiple Deprivation
ISPAD: International Society for Pediatric and Adolescent Diabetes
T1D: Type 1 diabetes
TW-HbAlc: Time-weighted glycated haemoglobin.

## Supplementary Materials

Supplementary Materials Figure S1: flowchart of patient inclusion. Figure S2: diabetic retinopathy (DR) cases over 15 years. Figure S3: socioeconomic deprivation and HbA1c by ethnicity. Table S1: characteristics of included and excluded patients. Table S2: age at diagnosis and DR screening details. Table S3: characteristics of patients with moderate or severe DR. Table S4: characteristics of patients with minimal or mild DR.
