# Supplementary material for: Rates, Risk Factors, and Progression of Diabetic Retinopathy in Children with Type 1 Diabetes: A 15-Year Retrospective Study from a Regional Center in New Zealand
Source: Pediatr Diabetes. 2024 Jun 28;2024:5893771. doi: 10.1155/2024/5893771 (PMC12020911; doi:10.1155/2024/5893771)
Supplement: Supplementary Materials — Figure S1: flowchart of patient inclusion. Figure S2: diabetic retinopathy (DR) cases over 15 years. Figure S3: socioeconomic deprivation and HbA1c by ethnicity. Table S1: characteristics of included and excluded patients. Table S2: age at diagnosis and DR screening details. Table S3: characteristics of patients with moderate or severe DR. Table S4: characteristics of patients with minimal or mild DR. [file 5893771.f1.docx]

**Supplementary Information**

**Rates, risk factors, and progression of diabetic retinopathy in children with type 1 diabetes: a 15-year retrospective study from a regional centre in New Zealand**

Thomas Winter^1^, José G B Derraik^2,3,4^, Craig A Jefferies^2,5,6^, Paul L Hofman^5,6^, James A D Shand^7^, Geoffrey D Braatvedt^7^, Stuti L Misra^1*^

^1^ Department of Ophthalmology, New Zealand National Eye Centre, Faculty of Medical and Health Sciences, University of Auckland, Auckland, New Zealand

^2^ Department of Paediatrics, Child and Youth Health, Faculty of Medical and Health Sciences, University of Auckland, Auckland, New Zealand

^3^ Environmental-Occupational Health Sciences and Non-Communicable Diseases Research Group, Research Institute for Health Sciences, Chiang Mai University, Chiang Mai, Thailand

^4^ Department of Women's and Children's Health, Uppsala University, Uppsala, Sweden

^5^ Starship Child Health, Health New Zealand | Te Whatu Ora Auckland, Auckland, New Zealand

^6^ Liggins Institute, University of Auckland, Auckland, New Zealand

^7^ Department of Endocrinology, Greenlane Clinical Centre, Health New Zealand | Te Whatu Ora, Auckland, New Zealand

***Author for correspondence:** Associate Professor Stuti L Misra; Department of Ophthalmology, New Zealand National Eye Centre, University of Auckland, Private Bag 92019, Auckland, New Zealand; email: [s.misra@auckland.ac.nz](mailto:s.misra@auckland.ac.nz)

**Figure S1**

**Diagram showing the number of children and adolescents with type 1 diabetes mellitus (T1D) in the Auckland region (New Zealand) screened for diabetic retinopathy and included in the 15-year audit (****2006–2020).**

**Figure S2**

**Cases of diabetic retinopathy (DR) among children and adolescents with type 1 diabetes mellitus (T1D) screened in the Auckland region over 15 years (2006–2020).**

A) Cumulative number of DR cases diagnosed.

B) Number of new DR cases diagnosed per year (grey bars; left *y*-axis) and number of DR screens recorded per year (black dots; right *y*-axis).

C) Scatter plot showing the linear association between the number of DR screens and new DR cases diagnosed per year; the simple linear regression line, its coefficients (*r*^2^), and *p*-value are also shown.

**Figure S3**

**Plots of socioeconomic deprivation and HbA1c by ethnicity among included patients with type 1 diabetes mellitus.**

**IMD18 panel** – Socioeconomic deprivation was estimated using the 2018 New Zealand Index of Multiple Deprivation (IMD18), with higher scores indicating greater deprivation (Exeter et al., PLoS ONE 2017;12:e0181260). Pairwise ethnic differences were examined using the Dwass, Steel, Critchlow-Fligner (DSCF) method to account for multiple comparisons (Douglas & Michael, Commun Statist Theory Meth 1991;20:127-139). Distinct capital letters above each ethnicity indicate statistically significant pairwise differences at *p*<0.001. Each box represents the interquartile range (IQR), defined by the first (Q1) and third (Q3) quartiles; the median is depicted as a line within the box, and the whiskers are the data range (smallest to largest values).

**HbA1c** – Glycated hemoglobin measured at the patient's first screening for diabetic retinopathy during the study period. All individual values are plotted, with the horizontal bars representing Q1, the median, and Q3. Pairwise differences were assessed using a general linear model with the Tukey-Kramer adjustment for multiple comparisons. Distinct capital letters above each ethnicity indicate statistically significant differences at *p*<0.01.

**Table S1**

**Demographic and clinical characteristics of children and adolescents with type 1 diabetes (T1D) in the Auckland region who were included or excluded from the study.**

| **Characteristic** | **Parameter** | **Levels** | **Included** | **Excluded** | ***p*-value** |
| --- | --- | --- | --- | --- | --- |
| ***n*** |  |  | 646 | 263 |  |
| **Demography** | **Age at T1D Dx (years)** |  | 7.4 ± 3.6 | 9.4 ± 4.4 | **<0.0001** |
|  |  |  | 7.2 [4.7, 10.5] | 10.3 [5.9, 13.4] | **<0.0001** |
|  |  |  | 0.7–14.7 | 0.7–15.9 |  |
|  | **Pubertal status at T1D Dx** | **Prepubertal** | 533 (82.5%) | 152 (57.8%) | **<0.0001** |
|  |  | **Pubertal** | 113 (17.5%) | 111 (42.2%) |  |
|  | **Age at clinic (years) †** |  | 9.6 ± 3.5 | 11.0 ± 4.6 | **<0.0001** |
|  | **IMD18 *** |  | 4 [2, 7] | 5 [2, 8] | 0.08 |
|  | **Sex** | **Female** | 302 (46.8%) | 124 (47.1%) | 0.94 |
|  |  | **Male** | 344 (53.3%) | 139 (52.9%) |  |
|  | **Ethnicity** | **NZ European** | 446 (69.0%) | 169 (64.3%) | **0.038** |
|  |  | **Māori** | 58 (9.0%) | 38 (14.4%) |  |
|  |  | **Pacific** | 55 (8.5%) | 28 (10.6%) |  |
|  |  | **Other** | 87 (13.5%) | 28 (10.6%) |  |
| **Anthropometry** | **Height SDS** |  | 0.58 ± 1.00 | 0.55 ± 1.04 | 0.70 |
|  | **Weight SDS** |  | 0.76 ± 0.94 | 0.82 ± 0.98 | 0.43 |
|  | **BMI SDS** |  | 0.67 ± 0.92 | 0.72 ± 0.94 | 0.48 |
| **Glycaemic control** | **HbA1c (mmol/mol)** |  | 62.5 ± 12.7 | 63.1 ± 17.1 | 0.58 |

Continuous data are the mean ± standard deviation, median [quartile 1, quartile 3], or the range; categorical data are *n* (%).

BMI, body mass index; Dx, diagnosis; HbA1c, glycated hemoglobin; IMD18, 2018 New Zealand Index of Multiple Deprivation; NZ, New Zealand; SDS, standard deviation score.

**^†^** First clinic recorded in the database within the study period covered by this study (2006–2020).

* The IMD18 is an area-based measure of socioeconomic deprivation where higher scores represent higher deprivation levels; data were available for 643 (99.5%) and 256 (97.3%) included and excluded participants, respectively.

*P*-values were derived from one-way ANOVA, non-parametric Kruskal-Wallis test, or Fisher’s exact tests; statistically significant *p*-values (at p<0.05) are highlighted in bold.

**Table S2**

**Age at type 1 diabetes mellitus diagnosis and the timing and number of diabetic retinopathy screens among included patients.**

| **Metrics** | **Statistics/Levels** | **Values** |
| --- | --- | --- |
| ***n*** |  | 646 |
| **Age at first DR screen (years)** | **Mean ± SD** | 12.6 ± 2.4 |
|  | **Median [Q1, Q3]** | 13.1 [10.9, 14.7] |
|  | **Range** | 5.9–16.4 |
| **Time from T1D diagnosis to first DR screen (years)** | **Mean ± SD** | 5.2 ± 2.2 |
|  | **Median [Q1, Q3]** | 5.2 [3.5, 6.2] |
|  | **Range** | 0.9–13.7 |
| **DR screens per patient (*n* )** | **1** | 180 (27.9%) |
|  | **2** | 175 (27.1%) |
|  | **3** | 105 (16.3%) |
|  | **4** | 85 (13.2%) |
|  | **5** | 48 (7.4%) |
|  | **6** | 29 (4.5%) |
|  | **7** | 17 (2.6%) |
|  | **8** | 5 (0.8%) |
|  | **9** | 1 (0.2%) |
|  | **10** | 1 (0.2%) |

Categorical data are expressed as *n* (%).

DR, diabetic retinopathy; Q1, quartile 1; Q3, quartile 3; T1D, type 1 diabetes mellitus.

**Table S3**

**Demographic and clinical characteristics of the only patients with type 1 diabetes mellitus (T1D) diagnosed with moderate or severe diabetic retinopathy (DR) during the study period.**

|  |  | **Retinopathy grade** | | | |
| --- | --- | --- | --- | --- | --- |
| **Timing** | **Parameter** | **Moderate** | **Moderate** | **Moderate** | **Severe** |
| **T1D diagnosis** | **Sex** | Male | Male | Female | Female |
|  | **Ethnicity** | NZ European | NZ European | Māori | NZ European |
|  | **Age (years)** | 10.1 | 14.2 | 6.0 | 8.1 |
|  | **IMD18 *** | 7 | 6 | 8 | 2 |
|  |  |  |  |  |  |
| **First screen** | **Age (years)** | 13.8 | 15.6 | 13.9 | 11.8 |
|  | **Time from T1D diagnosis to DR screen (years)** | 3.7 | 1.5 | 7.9 | 3.7 |
|  | **BMI SDS** | 2.22 | 0.82 | 1.05 | 1.00 |
|  | **HbA1c (mmol/mol)** | 73 | 74 | 83 | 63 |
|  | **Time-weighted HbA1c (mmol/mol)** | 65.8 | 62.8 | 81.6 | 68.0 |
|  |  |  |  |  |  |
| **At grade** | **Same as the first screen?** | No | Yes | No | No |
|  | **DR screen (order/total *n* )** | 4/4 | 1/1 | 2/2 | 2/2 |
|  | **Age (years)** | 16.9 | 15.6 | 15.1 | 13.1 |
|  | **Time from T1D diagnosis to DR screen (years)** | 6.9 | 1.5 | 9.2 | 5.1 |
|  | **BMI SDS** | n.a. | 0.82 | 1.07 | 0.24 |
|  | **HbA1c (mmol/mol)** | 65 | 74 | 79 | 64 |
|  | **Time-weighted HbA1c (mmol/mol)** | 66.9 | 62.8 | 81.4 | 68.2 |
|  |  |  |  |  |  |

BMI, body mass index; HbA1c, glycated hemoglobin; n.a., not available; NZ, New Zealand; SDS, standard deviation score.

* The IMD18 is an area-based measure of socioeconomic deprivation where higher scores represent greater deprivation levels (Exeter et al., PLoS ONE 2017;12:e0181260).

**Table S4**

**Demographic and clinical characteristics of children and adolescents with type 1 diabetes mellitus (T1D) in the Auckland region diagnosed with diabetic retinopathy (DR) at any time during the study period, for whom minimal or mild was the worst grade.**

| **Characteristic** | **Parameter** | **Levels** | **Minimal** | **Mild** | ***p-value*** |
| --- | --- | --- | --- | --- | --- |
| ***n*** |  |  | 209 | 146 |  |
| **Demography** | **Sex** | **Female** | 94 (45%) | 72 (49%) | 0.45 |
|  |  | **Male** | 115 (55%) | 74 (51%) |  |
|  | **Ethnicity** | **NZ European** | 144 (69%) | 91 (62%) | 0.52 |
|  |  | **Māori** | 17 (8%) | 12 (8%) |  |
|  |  | **Pacific** | 21 (10%) | 21 (14%) |  |
|  |  | **Other** | 27 (13%) | 22 (15%) |  |
|  | **IMD18 *** |  | 4 [2, 7] | 3 [5, 8] | **0.018** |
|  | **Height SDS** |  | 0.57 ± 0.96 | 0.29 ± 1.00 | **0.009** |
|  | **Weight SDS** |  | 0.76 ± 0.86 | 0.78 ± 0.98 | 0.85 |
|  | **BMI SDS (kg/m^2^)** |  | 0.62 ± 0.83 | 0.75 ± 0.92 | 0.19 |
| **Clinical** | **Age at T1D Dx (years)** |  | 7.0 ± 3.5 | 6.1 ± 3.2 | **0.023** |
|  |  |  | 6.8 [3.9, 10.0] | 6.2 [3.5, 8.4] |  |
|  |  |  | 0.8–14.1 | 0.7–13.6 |  |
|  | **Age at DR screen (years) ^2^** |  | 13.5 ± 2.1 | 13.8 ± 2.1 | 0.20 |
|  |  |  | 13.9 [12.1, 15.2] | 14.3 [12.9, 15.4] |  |
|  |  |  | 7.8–16.3 | 7.2–16.7 |  |
|  | **Time from T1D Dx to DR screen (years)** |  | 6.5 ± 2.7 | 7.2 ± 2.8 | **0.0001** |
|  | **HbA1c (mmol/mol)** |  | 70.5 ± 68.4 | 75.7 ± 17.2 | **0.003** |
|  | **Time-weighted HbA1c (mmol/mol)** |  | 66.5 ± 12.5 | 70.0 ± 14.5 | **0.002** |
|  | **DR screens (n)** | **1** | 35 (17%) | 15 (10%) | 0.51 |
|  |  | **2** | 44 (21%) | 32 (22%) |  |
|  |  | **3** | 46 (22%) | 31 (21%) |  |
|  |  | **4** | 38 (18%) | 26 (18%) |  |
|  |  | **5** | 35 (17%) | 15 (10%) |  |
|  |  | **≥6** | 44 (21%) | 32 (22%) |  |

Continuous data are the mean ± standard deviation, the median [quartile 1, quartile 3], or the range; categorical data are reported as n (%).

BMI, body mass index; HbA1c, glycated hemoglobin; IMD18, 2018 New Zealand Index of Multiple Deprivation; NZ, New Zealand; SDS, standard deviation score.

* The IMD18 is an area-based measure of socioeconomic deprivation where higher scores represent higher deprivation levels; data were available for all patients with minimal DR and 98.6% (144/146) of those with mild DR.

† First screen recorded in the database that met the inclusion criteria for this study.

*P*-values were derived from one-way ANOVA, non-parametric Kruskal-Wallis tests, or Fisher’s exact tests; statistically significant *p*-values (at *p*<0.05) are highlighted in bold.
